# Re-evaluation of indications and outcomes of endoscopic excision procedures for colorectal tumors: a review

**DOI:** 10.1093/gastro/got034

**Published:** 2013-12-24

**Authors:** Shilun Cai, Yunshi Zhong, Pinghong Zhou, Jianmin Xu, Liqing Yao

**Affiliations:** ^1^Endoscopic Center, Zhongshan Hospital of Fudan University, Shanghai, China and ^2^Department of General Surgery, Zhongshan Hospital of Fudan University, Shanghai, China

**Keywords:** Colorectal cancer, endoscopic excision, endoscopic mucosal resection, endoscopic submucosal dissection

## Abstract

Endoscopic submucosal dissection (ESD) and endoscopic mucosal resection (EMR) are useful therapeutic techniques for colorectal tumors. Currently, new techniques based on these procedures are available, such as endoscopic submucosal dissection with snare (ESD-S) and endoscopic mucosal resection with pre-cutting (EMR-P). For the excision of colorectal tumors, each of these techniques has been characterized as having a high total resection rate, low recurrence rate or low complication rate. In this study, we analysed clinical trials that had recently been published, to search for the most appropriate endoscopic treatment for colorectal tumors. Our search results revealed the following: for a tumor with a diameter less than 20 mm, the surgeon should choose ESD, ESD-S, EMR-P or EMR, depending on the condition of the tumor. On the other hand, to excise a tumor larger than 20 mm in diameter, ESD and ESD-S should be the first choices. However, if the patient has a high risk of complications due to ESD or ESD-S, the use of EMR-P would be suitable. Because of the high possibility of canceration in a tumor larger than 20 mm in diameter, EMR is not the optimal endoscopic treatment for the excision of a colorectal tumor, due to a low total resection rate and a high recurrence rate.

## INTRODUCTION

Endoscopic submucosal dissection (ESD) and endoscopic mucosal resection (EMR) are two different types of digestive endoscopic surgical procedures. Both of these procedures have the features of being non-aggressive and resulting in mild postoperative pain, quick recovery, etc.; therefore, they can be used for minimally invasive treatment of early gastrointestinal tumors. EMR has been widely recognized and has become a routine method for the treatment of early gastrointestinal mucosal lesions [1]. However, EMR generally does not apply to lesions of more than 20 mm in diameter, and studies have shown that the overall block- and total resection rates were only 42.9% and 32.9%, respectively [2]. ESD stems from the development of EMR, which was named after 2003 and approved as a new treatment modality. The emergence of the insulation-tipped knife (knife IT) marked the point when endoscopic treatment entered the ESD era. ESD can completely strip entire large lesions, providing a low rate of recurrence. However, the demands on the equipment and operating personnel are relatively high and the incidence of complications correlates with the operator’s technical proficiency [3]. With their own characteristics, ESD and EMR can both be selectively used in the treatment of gastrointestinal mucosal and submucosal lesions.

At present, two new methods, ESD with snare (ESD-S) and EMR with pre-cutting (EMR-P), are becoming more common, as they are improved versions of ESD and EMR. In this paper, we review the applications of ESD, EMR, ESD-S, and EMR-P in the treatment of colorectal cancers. The colorectal cancers analysed in this article include mucosal source polyps, as well as early carcinomas (tumor node metastasis stage TisN0M0 to T1N0M0 colorectal cancers).

## SURGICAL PROCEDURES, INDICATIONS, AND CONTRAINDICATIONS FOR EMR AND EMR-P

### Surgical procedures for EMR and EMR-P

**Figure 1.**
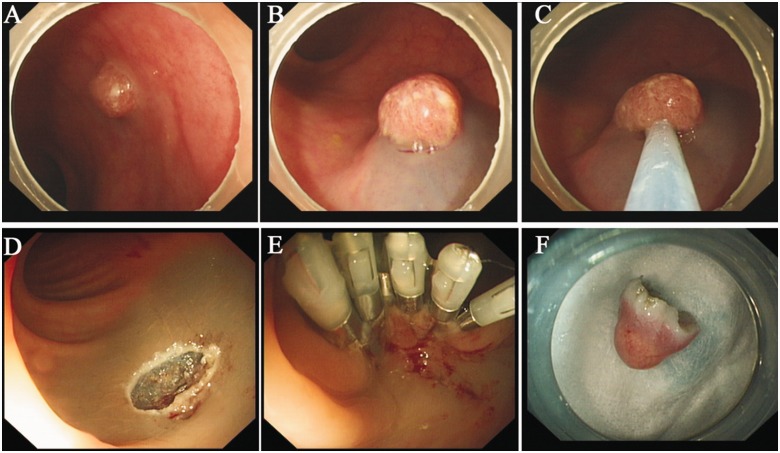
The process of endoscopic mucosal resection (EMR). (A) The lesion before resection. (B) Inject saline solution at the submucosa. (C) Release the snare, then re-tighten and resect the lesion. (D) The wound after resection. (E) Seal the wound with metallic clips. (F) The lesion.

The surgical procedure for EMR is as follows (Figure 1):

1. Tags: use argon plasma coagulation (APC) for electric coagulation markers at 0.5 cm from the edge of the lesion.
2. Injection: perform submucosal multi-point injections of 1:100,000 adrenaline saline solution at the lateral end of the marked points, to make the lesion and its surrounding tissues swell (positive lifting sign), and add methylene blue when necessary.
3. Install the transparent cap at the front-end of the endoscope.
4. Insert the endoscope again: place the snare at the inner edge of the transparent cap, fully fasten the lesion tissues into the transparent cap by negative pressure attraction, release and then re-tighten the snare, and resect the lesions.
5. Manage the wound surface: use hot biopsy forceps, clamps and electric coagulation for the small blood vessels visible at the wound surface, spray local hemostasis and anti-infective drugs, and apply a titanium clamp to partially or completely close the wounds when necessary [4].

The procedure for EMR-P is roughly the same as for EMR (figures are not provided, since this procedure has not yet been carried out in our endoscopic center). The difference in the procedure for EMR-P is that, after making the electric coagulation markers, the operator first cuts a circle along the marked points around the lesion, and then makes the snare overlap with the ring tangent, tightens the snare after confirming that all the tumor tissues have entered into the snare, and finally removes the tumor tissues [5].

### Indications

In theory, the indications for an EMR operation include that the lesions have a definite pre-operative diagnosis by endoscopy and histological examination, that they originated from the mucosa, that an integrated local bowel wall structure is present, and that there is no evidence of enlarged lymph nodes or lymph node metastasis. It is generally recognized that submucosal tumors originating from the gastrointestinal submucosa can also be treated with an EMR operation, while lesions derived from the muscularis propria or serosa are usually treated by surgery or laparoscopy [6]. The EMR-P operation is suitable for situations in which the tumor is larger than the snare or if the tumor location is not convenient for the traditional EMR operation [3]. The EMR technique is applied to small lesions, while larger ones (greater than 20 mm) have been gradually treated by ESD or EMR-P overseas [7]. However, the application of EMR and ESD techniques varies in different areas of China.

### Contraindications

The contraindications for EMR and EMR-P operations are the same as those for routine digestive endoscopic procedures.

## SURGICAL PROCEDURES, INDICATIONS, AND CONTRAINDICATIONS FOR ESD AND ESD-S

### Surgical procedures for ESD and ESD-S

**Figure 2.**
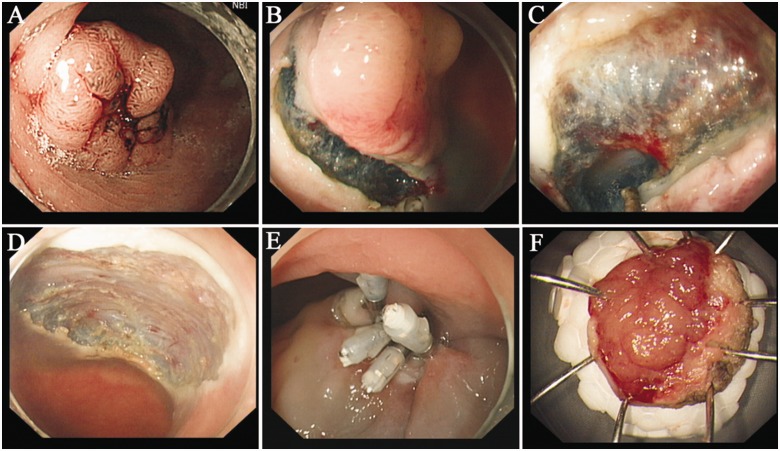
The process of endoscopic submucosal dissection (ESD). (A) A narrow-band image showing the edges of the lesion. (B) Inject saline solution at the submucosa and cut the lesion. (C) Remove the lesion at the bottom. (D) The wound after resection. (E) Seal the wound with metallic clips. (F) The lesion.

The surgical procedures for the ESD operation are as follows (Figure 2):

1. Tags: use the indigo carmine or narrow-banding imaging (NBI) stain to show the edges of the lesions and apply an argon knife to make electric coagulation markers at the edge of the apophysis lesions.
2. Injection: apply multi-point injections of saline solution (containing indigo carmine and adrenaline) into the submucosal tissue at lateral areas of the mark points. The injection of a small amount of indigo carmine and adrenaline (generally mixed using 100 ml of saline, 5 ml of 0.8% indigo carmine, and 1 ml of adrenaline) can significantly improve the effect of display and function, as the indigo carmine can make the submucosa and muscularis better defined and cause submucosal injection areas to be displayed more clearly, while adrenaline can shrink the smaller blood vessels and thus reduce bleeding. The operator should inject some hyaluronic acid if possible, which can prolong apophysis, revealing the edge of the lesion more clearly, and reducing time wasted due to repeated injections, as compared with normal saline.
3. Cut the edge: use an IT or Hook knife to cut the mucosa along the marked points.
4. Strip lesions: apply an IT or Hook knife to strip the submucosa at the bottom of the lesions [8, 9].

**Figure 3.**
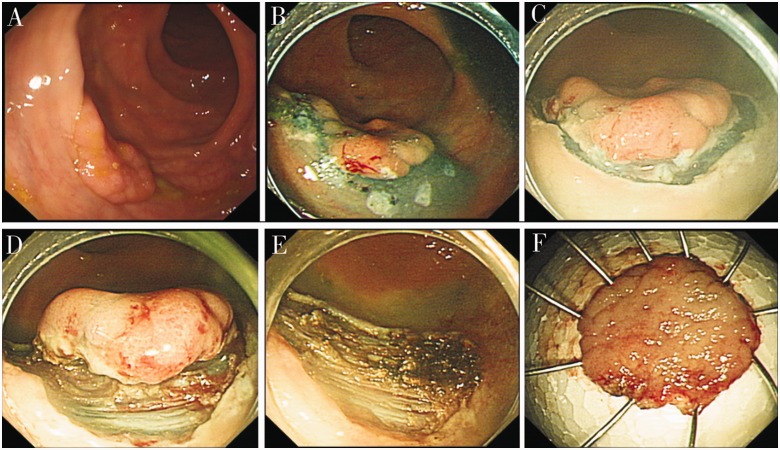
The process of endoscopic submucosal dissection with snare (ESD-S). (A) The lesion before resection. (B) Inject saline solution at the submucosal at the lateral of the marked multi-points. (C) Cut the edge of lesion. (D) Use snare to resect lesion. (E) The wound after resection. (F) The lesion.

The procedure for ESD-S is approximately the same as that for ESD (Figure 3). The difference with the ESD-S technique is that the operator switches to using a snare to completely separate the remaining tissue directly after the lesions have been reduced to a quarter or even smaller proportion of their total size, instead of using an IT or Hook knife to resect them.

### Indications

According to the 2010 guidelines on ESD technique in the treatment of colorectal cancer in Japanese patients (Table 1), the indications can be roughly divided into the following four types: i) tumors greater than 20 mm in diameter, which are difficult to remove by an EMR operation; ii) tumors originating from the mucous membrane that are associated with fibrosis, mainly caused by biopsy or lesion prolapse as intestinal peristalsis; iii) scattered tumors caused by chronic inflammation and iv) the incomplete resection of early tumors by endoscopic surgery [11]. In addition, it has been reported that rectal tumors of less than 10 mm diameter can be safely and neatly resected by an EMR operation, and thus these tumors are not indications for an ESD operation [12]. In China, the application of ESD in the treatment of early gastric cancers has been widely accepted. However, the application of ESD to the treatment of large intestinal cancers is not yet widely carried out. At present, early colorectal cancer can be treated by radical local excision through an ESD operation, as long as the disease is confined to the superficial mucosa, since the risk of lymph node metastasis for such early colorectal cancer is less than 1% [13, 14].

**Table 1.** Indication criteria for the ESD operation in the treatment of colorectal cancer

Tumors greater than 20 mm diameter conform to the indication of endoscopic treatment but are difficult using the EMR operation: The grain-type of laterally spreading tumor of the large intestine (LST-NG), especially for the pseudo-depressed type Pathology-revealed type VI tumors with gland opening Tumors with submucosal invasion Larger umbilicate-type tumors Larger lesions suspected as tumors Mucosal tumors with fibrosis (lesions prolapse mainly caused by biopsy or intestinal gurgling) Scattered tumors caused by chronic inflammation, such as cancer caused by ulcerative colitis Residual early tumor lesions after endoscopic operation

ESD-S is applicable to tumors with a higher risk of bleeding perforation, such as severe inflammation of the mucous membrane layer or submucosa. Meanwhile, the ESD-S method can be used to remove residual lesions, if there is intra-operative bleeding or perforation appearing at the time of the ESD operation [15, 16].

### Contraindications

The contraindications for ESD and ESD-S operations are the same as those for routine digestive endoscopic procedures.

## CLINICAL OUTCOMES: TOTAL RESECTION RATE AND POST-OPERATIVE EVALUATION

The definition of total resection is that the lesion is removed as a single piece in a one-time excision, and there is no residual tissue, either at the edge of the removed tissue samples or at the bottom of the lesion [17]. The comparison between several types of endoscopic surgery in the treatment of colorectal cancer, found in the literature reports of recent years, is summarized in Tables 2 and 3.

**Table 2.** Total resection and recurrence rates for ESD, EMR, ESD-S, and EMR-P (tumor diameter <20 mm)

Total number	Modality/ number	Total resection rate	*P* value	Recurrence rate	*P* value	Reference	Published year
74	ESD/46	82.6%	0.067	0.0%	0.378	Lee, D.S.^[^^18^^]^	2010
	EMR/28	64.3%		3.6%^a^			
79	ESD/8	87.5%	0.292	0.0%	NS	Kim, Y.J.^[^^3^^]^	2013
	ESD-S/20	60.0%		0.0%			
	EMR-P/51	58.8%		0.0%			
75	ESD/44	97.7%	0.007	0.0%	NS	Kim, K.M.^[^^19^^]^	2013
	EMR/31	77.4%		0.0%			

NS = no significant difference.

^a^One patient relapsed *in situ* 10 months after surgery.

**Table 3.** Total resection and recurrence rates of ESD, EMR, ESD-S, and EMR-P (tumor diameter >20 mm)

Total number	Modality/ number	Total resection rate	*P* value	Recurrence rate	*P* value	Reference	Published year
127	ESD/50	74.0%	0.007	0.0%	NS	Kim^[^^3^^]^	2013
	ESD-S/37	51.4%		0.0%			
	EMR-P/40	42.5%		0.0%			
523	ESD/314	87.6%	ESD vs EMR<0.001	0.8%	ESD vs EMR<0.001	Lee^[^^2^^]^	2012
	EMR/140	32.9%	ESD vs EMR-P = 0.006	25.7%	ESD vs EMR-P = 0.303		
	EMR-P/69	59.4%	EMR-P vs EMR<0.001	3.1%	EMR-P vs EMR<0.001		
189	ESD/85	83.5%	<0.001	1.0%	0.002	Tajika^[^^20^^]^	2011
	EMR/104	48.1%		16.0%			
159	ESD/61	91.8%	–	0.0%	–	Terasaki^[^^21^^]^	2012
	ESD-S/28	96.4%		0.0%			
	EMR/70	98.6%		1.4%			
84	ESD/28	–	–	0.0%	0.008	Kobayashi^[^^22^^]^	2012
	EMR/56	–		21.4%			

### Tumors with a diameter less than 20 mm

Lee *et al.* reported that there were no statistical differences, between ESD and EMR groups, in the total resection and recurrence rates for tumors less than 20 mm [18]. Kim Y.J. *et al.* discovered that there was no statistical difference in the resection rates between EMR-P, ESD, and ESD-S, and no recurrence in the three groups [3]. Kim K.M. *et al.* reported that there was a statistical difference in the total resection rates between the ESD and EMR groups (*P* = 0.007), but no patients relapsed in either group [19]. Of these three studies, the pathological tumor type in the reports by Lee *et al.* and Kim K.M. *et al.* was adenoma without early adenocarcinoma [18, 19], while Kim Y.J. *et al.* [3] did not provide detailed pathological classification data.

### Tumors larger than 20 mm diameter

Kim Y.J. *et al.* reported that there were statistical differences in the total resection rates between ESD, ESD-S, and EMR-P groups for tumors larger than 20 mm in diameter (*P* = 0.007), and no patients relapsed in the three groups [3]. Lee *et al.* reported that there were statistical differences in the total resection rates between ESD, EMR, and EMR-P treatments, and there were significant differences in the recurrence rates between ESD and EMR, and between EMR-P and EMR, but no difference found between ESD and EMR-P groups [2]. Tajika *et al.* reported that there were statistical differences in the total resection- and recurrence rates between ESD and EMR groups [20].

Except for the study by Kim *et al.* [3], the studies included in Tables 2 and 3 did not provide detailed data on the pathological tumor types; the proportions of adenocarcinoma in other research studies were as follows: 30.78% (161/523) of Lee *et al.* [2], 40.74% (77/189) of Tajika *et al.* [20], 41.51% (66/159) of Terasaki *et al.* [21], and 71.43% (60/84) of Kobayashi *et al.* [22], and other sources of tumors were less than 2%. There were no statistical differences between the proportions of pathological tumor types between the different operations in all studies. The invasion scopes of colorectal cancer were all limited to the mucosa or the submucosa, with tumor node metastasis stage TisN0M0 to T1N0M0 and clinical stage 0 to stage 1, and the tumors were located from the right colon to the rectum.

## MAJOR INTRA-OPERATIVE COMPLICATIONS: HEMORRHAGE AND PERFORATION

The main complications of ESD and EMR are bleeding and perforation. The causes of complications include mainly the thinness or fragile texture of the colorectal intestinal wall, partial fibrosis of the submucosa, the disease situation of the patients, and improper methods used by the operator [23]. Comparisons of the intra-operative complications between several surgical procedures in recent years are shown in Tables 4 and 5.

**Table 4.** Complications following ESD, EMR, ESD-S, and EMR-P (tumor diameter <20 mm)

Total number	Modality/ number	Bleeding rate	*P* value	Perforation rate	*P* value	Complication *P* values	Reference	Published year
74	ESD/46	4.3%	–	2.2%	–	0.586	Lee^[^^18^^]^	2010
	EMR/28	3.6%		0.0%				
79	ESD/8	0.0%	0.681	12.5%^a^	0.244	–	Kim^[^^3^^]^	2013
	ESD-S/20	5.0%		15.0%				
	EMR-P/51	2.0%		3.9%				
75	ESD/44	0.0%	NS	0.0%	NS	NS	Kim^[^^19^^]^	2013
	EMR/31	0.0%		0.0%				

NS = no significant difference.

^a^One patient had perforation during the operation.

**Table 5.** Complications following ESD, EMR, ESD-S, and EMR-P (tumor diameter >20 mm)

Total number	Modality/ number	Bleeding rate	*P* value	Perforation rate	*P* value	Complications *P* value	Reference	Published year
127	ESD/50	10.0%	0.069	16.0%	0.214	–	Kim^[^^3^^]^	2013
	ESD-S/37	13.5%		21.6%				
	EMR-P/40	0.0%		7.5%				
523	ESD/314	0.6%	–	8.0%	ESD vs EMR<0.001	ESD vs EMR = 0.024	Lee^[^^2^^]^	2012
	EMR/140	0.0%		0.0%	ESD vs EMR-P = 0.048	ESD vs EMR-P = 0.348		
	EMR-P/69	2.9%		2.9%	EMR-P vs EMR = 0.321	EMR-P vs EMR = 0.038		
189	ESD/85	2.4%	NS	5.9%	0.040	–	Tajika^[^^20^^]^	2011
	EMR/104	2.9%		0.0%				
159	ESD/61	11.5%	NS	0.0%	NS	–	Terasaki^[^^21^^]^	2012
	ESD-S/28	0.0%		7.1%				
	EMR/70	7.1%		0.0%				
84	ESD/28	7.1%	0.2	10.7%	0.008	–	Kobayashi^[^^22^^]^	2012
	EMR/56	1.8%		0.0%				

### Tumors of less than 20 mm diameter

Lee *et al.* reported that the bleeding and perforation rates of the ESD group were 4.3% and 2.2%, respectively [18]; the hemorrhage rate of the EMR group was 3.6%; and there were no cases of perforation. Thus, the total numbers of complications between the two groups were not significantly different (*P* = 0.586). Kim *et al.* [3] reported the comparisons between ESD, ESD-S, and EMR-P; the hemorrhage rates of the three groups were 0%, 5%, and 2% (*P* = 0.681), respectively, and the perforation rates were 12.5%, 15%, and 3.9% (*P* = 0.244), respectively. No complications occurred in the two groups reported by Kim *et al.* [19].

### Tumors greater than 20 mm diameter

Kim *et al.* [3] reported that the bleeding rates after ESD, ESD-S, and EMR-P were 10.0%, 13.5%, and 0% (*P* = 0.069), respectively, and the perforation rates were 16%, 21.6%, and 7.5% (*P* = 0.214), respectively. Lee *et al.* reported that the bleeding and the perforation rates of the ESD group were 0.6% and 8%, respectively [2], while no patients had bleeding or perforation in the EMR group and the bleeding and perforation rates of the EMR-P group were both 2.9%. For the perforation rate, there were statistical differences for ESD vs EMR (*P* < 0.01) and ESD vs EMR-P (*P* = 0.048), while there was no statistical difference for EMR-P vs EMR (*P* = 0.321). For the total complications, there were no statistical differences for ESD vs EMR (*P* = 0.024), ESD vs EMR-P (*P* = 0.348), or EMR-P vs EMR (*P* = 0.038). Tajika *et al.* [20] reported the comparison results of ESD and EMR; the bleeding rates of the two groups were 2.4% and 2.9%, respectively (*P* > 0.05), while the perforation rates were 5.9% and 0%, respectively (*P* = 0.040). The results of Terasaki *et al.* [21] revealed that the respective bleeding and perforation rates were 11.5% and 0% for the ESD group, 0% and 7.1% for the ESD-S group, 7.1% and 1.4% for the EMR group, and there were no statistical differences for either the bleeding or the perforation rates. Kobayashi *et al.* reported that the bleeding rates of the ESD and EMR groups were 7.1% and 1.8% (*P* = 0.2) [22], and the perforation rates of the two groups were 10.7% and 0%, respectively (*P* = 0.013).

## CLINICAL OUTCOMES AND COMPLICATIONS OF EMR AND ESD FROM SINGLE-METHOD STUDIES

Lee *et al.* performed the ESD technique in 1000 cases [24]; the total resection rate was 91.2%, the recurrence rate was 0.4% and the bleeding and perforation rates were 0.4% and 5.3%, respectively. Meanwhile, the research of Saito *et al.* [25] included 1321 cases of ESD data at 11 centers; the total resection rate was 87.2%, the hemorrhage rate was 2.5%, and the perforation rate was 2.9%. The recurrence rate was not provided. In contrast to the ESD technique, the number of cases who received the EMR operation was relatively low. Park *et al.* carried out statistical analysis of 236 cases who underwent the EMR technique [26]; they found that the total resection rate was 68.6%, the recurrence rate was 0.8%, and the bleeding and perforation rates were 8.1% and 1.3%, respectively. In addition, Serrano *et al.* included 140 cases of EMR in their study [27]; their results showed that the total resection rate was 91.4%, the recurrence rate 18.9% and the hemorrhage and perforation rates 5.0% and 0.7%, respectively.

## COMPREHENSIVE ANALYSIS

1. In dealing with a colorectal tumor under 20 mm in diameter, EMR has a less favorable total resection rate, compared with ESD, ESD-S and EMR-P, but no difference exists concerning the complication and recurrence rates between the four different operations. After calculating the RR value through the STATA software and integrating the results provided in Tables 2 and 4, it was found that, in dealing with tumor lesions less than 20 mm in diameter, there were no statistical differences between EMR and ESD in the bleeding rate (*P* = 0.870), perforation rate (*P* = 0.703) or recurrence rate. According to the results of Kim *et al.* [3], there were no statistical differences between the ESD-S, EMR-P, EMR, and ESD groups in the bleeding rate (*P* = 0.681), perforation ra*t*e (*P* = 0.244), or recurrence rate. As for the total resection rate, the ESD group had a significantly greater rate than that of the EMR group (*P* = 0.007), while no statistical differences were found between the ESD-S, EMR-P, and ESD groups (*P* = 0.292) in the treatment of small tumors.
2. As for tumors larger than 20 mm in diameter:
  - ESD and ESD-S had similar total resection rates, complications, and recurrence rates.
  - The EMR operation is not recommended for resection of early colorectal carcinoma: the EMR technique is superior to the ESD technique in the treatment of colorectal benign lesions, since it has a low risk of complications, a short operation time, and the characteristics of the operation method are relatively simple; thus, the effect of EMR is equivalent to or better than that of ESD, even for large, benign tumors [28, 29]. As early colorectal carcinoma has characteristics of invasion, recurrence, and metastasis and the organization of the tumor site may change, it is very important to mandate complete resection. According to the reports described in Table 5, the proportion of adenocarcinoma significantly increases in tumors over 20 mm in diameter, since of the degree of tumor malignancy is often associated with tumor size. It has been reported that the size of the tumor can be partly used as an index to predict the degree of malignancy, since the possibility of recurrence of tumors greater than 10 mm diameter has been shown to be relatively high [30], while the risk of lymph node metastasis was shown to be less than 3% for tumors less than 10 mm in diameter [18]. As the possibility of cancer progression is greater for a tumor larger than 20 mm and since the data demonstrated that the total resection and recurrence rates of the EMR technique were not ideal, we concluded that there are limitations for applying the EMR technique in the treatment of early colorectal cancer.
  - The total resection rate of EMR-P is less than that of ESD but with a similar recurrence rate and a significantly reduced incidence of complications. Therefore, when performing the pre-operative assessment, the surgeon should pay attention to the patient’s intestinal wall and observe whether it is too weak or has a brittle texture. In addition, the surgeon should pay attention to the degree of inflammation of the tumor. If the he/she considers that the risks of ESD and ESD-S are great, or that the location of the tumor is difficult for the operation, the EMR-P operation may be adopted.

The results of the data analysis were as follows:

As for the aspects of bleeding rate, there were no statistical differences between the four operation techniques (ESD vs EMR, *P* = 0.180; ESD-S vs ESD, *P* = 0.509; EMR-P vs ESD, *P* = 0.694). In terms of the perforation rate, the ESD group had an obviously greater rate than that of the EMR group (*P* = 0.001), while there were no differences between the ESD, ESD-S, and EMR-P groups (ESD vs ESD-S, *P* = 0.166; ESD vs EMR-P, *P* = 0.067). The reason for the greater perforation rate of the ESD-S and ESD groups may be due to the fact that ESD-S and ESD are more prone to resulting in perforation than EMR when dealing with tumor tissue, as they are likely to be affected by the *modus operandi* of the ESD technique itself. The reason for more complications with EMR-P than with EMR may be due to the fact that most lesions chosen for this operation are larger and they are prone to perforations during the pre-cutting step.

The comparative results of the total resection and recurrence rates between the four different procedures are as follows:

The total resection rate of ESD was significantly greater than that of EMR (*P* < 0.001), while the recurrence rate was significantly less than that of EMR (*P* < 0.001). Compared with EMR-P, ESD had an obviously greater total resection rate (*P* < 0.001), but no statistical difference existed for the recurrence rate (*P* = 0.219). In addition, there were no differences between EMR-P and ESD in the total resection or recurrence rates (*P* = 0.124; *P* = NS). Likewise, there were no statistical differences between EMR-P and ESD-S in either the total resection or recurrence rates, (*P* = 0.438, *P* = NS).

Table 6 displays some single method studies about EMR or ESD, from which we obtained results similar to those above. In the treatment of colorectal cancer, the ESD technique has the characteristics of a high total resection rate and a low recurrence rate compared with EMR, but ESD also has a greater risk of perforation when compared with the EMR operation.

**Table 6.** Clinical outcomes and complications following EMR and ESD, from single method studies

Total number	Operative procedure	Total resection rate	Recurrence rate	Bleeding rate	Perforation rate	Reference	Published year
1000	ESD	91.2%	0.4%	0.4%	5.3%	Lee^[^^24^^]^	2013
1321	ESD	87.2%	-	2.5%	2.9%	Saito^[^^25^^]^	2012
236	EMR	68.6%	0.8%	8.1%	1.3%	Park^[^^26^^]^	2011
140	EMR	91.4%	18.9%	5.0%	0.7%	Serrano^[^^27^^]^	2012

## SUMMARY

In conclusion, there are several different kinds of endoscopic surgery available for the treatment of colorectal cancer. ESD, ESD-S, and EMR-P have significant advantages in terms of their total resection rates and recurrence rates in treating smaller tumors (under 20 mm in diameter). Due to the small size of the lesions, there are no significant differences between the three methods and EMR, in terms of complication risk. As the risk of cancer progression for small tumors is less and the main pathological type is adenoma, EMR can be used for lesions with a relatively smooth surface and without bleeding and erosion, according to the microscopic analysis with close follow-up, although the total resection rate of EMR is low. For larger tumors (greater than 20 mm diameter), EMR is not recommended because the possibility of cancer development is greater, and the total resection and recurrence rates of EMR are not ideal. Due to their higher total resection rates and lower recurrence rates, ESD and ESD-S can be considered as the first choice for such tumors. However, it is important to note that, as the incidence of complications relating to ESD and ESD-S was obviously increased, the pre-operative assessment should pay attention to the patient’s intestinal wall, observing whether it is too weak or has a brittle texture, and also to the degree of inflammation of the tumor. If the risk is assessed as great, or the tumor location creates difficulty when using the ESD or ESD-S techniques, EMR-P might be adopted. Although the total resection rate of EMR-P is lower, the recurrence rate is not significantly different from the two former methods. Using the EMR-P technique in this situation can reduce the risk of bleeding and perforation, as well as reducing the possibility of repeated surgical repair.

## FUNDING

The National Natural Science Foundation of China (Grant No. 81101566).

**Conflict of interest:** none declared.
